# Efficacy of dietary supplements targeting gut microbiota in the prevention and treatment of gestational diabetes mellitus

**DOI:** 10.3389/fmicb.2022.927883

**Published:** 2022-07-14

**Authors:** Jiayang Wan, Jingmei Ma

**Affiliations:** Department of Obstetrics and Gynecology, Peking University First Hospital, Beijing, China

**Keywords:** probiotics, prebiotics, gut microbiota, gestational diabetes mellitus, obesity, type 2 diabetes mellitus

## Abstract

Gestational diabetes mellitus (GDM) is a kind of metabolic disease occurring during gestation period, which often leads to adverse pregnancy outcomes and seriously harms the health of mothers and infants. The pathogenesis of GDM may be bound up with the abnormal gut microbiota composition in pregnant women. Previous studies have clarified that dietary supplements can regulate the gut microbiota to play a role. Therefore, using dietary supplements, such as probiotics, prebiotics, and synbiotics to target the gut microbiota to regulate the disordered gut microbiota would become a potential method that benefits for preventing and treating GDM. This paper reviews a series of clinical trials in recent years, expounds on the clinical effects of dietary supplements such as probiotics on GDM, and discusses the intervention effects of dietary supplements on GDM related risk factors, including overweight, obesity, and type 2 diabetes mellitus (T2DM). In addition, the relationship of GDM and gut microbiota is also discussed, and the possible mechanisms of dietary supplements are summarized. This review will help to promote the further development of dietary supplements targeting gut microbiota and provide more knowledge support for clinical application in the prevention and treatment of various diseases.

## Introduction

Gestational diabetes mellitus (GDM) is discovered in pregnancy firstly, which is a metabolic disorder during pregnancy with impaired glucose tolerance (Baz et al., 2016). The imbalance of gut microbiota is related to the formation of GDM (Hasain et al., 2020). In the past few decades, the prevalence of GDM has been increasing, the prevalence of GDM in the United States is estimated to be 4.6–9.2% (DeSisto et al., 2014), the reported prevalence of GDM in China is 9.3–18.9% (Gao et al., 2019). The global prevalence of hyperglycaemia in pregnancy is 15.8%, and the global prevalence of GDM is 12.8% (Yuen et al., 2019). GDM also leads to long-term risk in pregnant women and infants such as type 2 diabetes mellitus (T2DM; Song et al., 2018), affecting the health of mothers and offspring. A large meta-analysis and systematic review showed that women with a history of GDM had a 10-fold higher risk of developing T2DM, especially in the 5 years after delivery (Vounzoulaki et al., 2020). The Hyperglycemia and Adverse Pregnancy Outcome (HAPO) follow-up studies found that more than 50% of women diagnosed with GDM had impaired glucose tolerance after pregnancy (Lowe et al., 2018). Therefore, with the increasing incidence of GDM and the consequent occurrence of T2DM, GDM needs more attention. GDM has multiple risk factors. Maternal obesity and overweight are essential adverse factors for developing GDM (Poston et al., 2016). Pregnant women’s weight gain during pregnancy and pre-pregnancy weight gain are closely related to GDM (Najafi et al., 2019; Lan et al., 2020). Gestational women with a family history of T2DM were more likely to have a higher incidence of GDM (Solomon et al., 1997; Chen et al., 2021). Therefore, it is necessary to find effective interventions to prevent and treat GDM and its related risk factors.

As a pregnancy metabolic disease, GDM is associated with insulin resistance (Baz et al., 2016). The imbalance of gut microbiota is related to the pathogenesis of GDM (Wang et al., 2020). In recent years, the imbalance of gut microbiota is considered a vital reason leading to the occurrence of metabolic diseases (Fan and Pedersen, 2021). Gut microbiota homeostasis can be considered an important part of the balance of the whole metabolic system. Regulating beneficial bacteria and conditional pathogens in the intestine is helpful to improve metabolic function (Lin and Zhang, 2017). Consequently, targeted regulation of gut microbiota to improve metabolism is a possible way (Guevara-Cruz et al., 2019). At present, the main dietary supplements interventions include probiotics, prebiotics, and synbiotics, which can target gut microbiota through various mechanisms, such as improving intestinal barrier function (La Fata et al., 2018) and regulating immunity (Vulevic et al., 2015). Commonly used probiotics include *Lactobacillus* and *Bifidobacterium* (Azad et al., 2018), which are used in various dosage forms. Common prebiotics include galactooligosaccharides (GOS), fructooligosaccharides (FOS), inulin, and some dietary fibers (Gibson et al., 2017).

Recently, the intake of dietary supplements to interfere with diseases by targeting gut microbiota has become a popular research direction. This paper reviews some research status of the efficacy of the above intervention measures in the prevention and treatment of GDM and related diseases, providing more evidence for the clinical application of dietary supplements.

## GDM and gut microbiota

In the first and second trimesters, as gestational weeks increase, the fetal demand for nutrients increases, and glucose acquisition from the mother through the placenta becomes the primary source of energy for the fetus (Di Cianni et al., 2003). Maternal plasma glucose levels decrease as pregnancy progresses, with approximately a 10% reduction in fasting plasma glucose (FPG). By mid to late gestation, there is an increase in antagonistic insulin-like substances, such as tumor necrosis factor (TNF), leptin, placental lactogen, estrogen, progesterone, and cortisol (Powe et al., 2019, 2022). Insulin resistance exacerbates β-cell dysfunction, reduced glucose uptake further leads to hyperglycemia, and β-cell overburden requires the production of additional insulin as feedback. As a result of impaired metabolism, glycogen accumulation contributes to this “glucotoxicity” by dysregulating the biochemical pathways that promote β-cell dysfunction (Ashcroft et al., 2017). The sensitivity of pregnant women to insulin decreases as gestational age increases. In order to maintain the normal level of glucose metabolism, insulin requirements must increase accordingly (Di Cianni et al., 2003). However, for women with restricted insulin secretion, the gestation status does not alleviate this metabolic change, so GDM occurs, or the degree of preexisting diabetes increases.

Some studies indicate that gut microbiota participates in the pathogenesis of GDM (Kuang et al., 2017; Wang et al., 2018). The human microbiota plays a crucial role in health. In general, the gut microbiota remains relatively stable during normal human pregnancy (DiGiulio et al., 2015). But it has also been shown that the composition of gut microbiota changes significantly at different stages of pregnancy, such as the decrease of butyrate-producing bacteria, the overall increase of *Proteobacteria* and *Actinobacteria*, the reduction of flora richness and α diversity, and β diversity increasing at the late pregnancy (Koren et al., 2012). A small number of studies looked at the microbiota of GDM patients and showed opposite results, with no differences (Koren et al., 2012). Kuang et al. (2017) found that the abundance of bacteria in GDM patients and healthy controls was different at the genus level. Compared with average blood glucose in pregnant women, the gut microbiota of GDM was abnormal at phylum and genus levels. In the GDM cohort, the abundance of *Actinomycetes* at the phylum level and *Collinella*, *Rothia*, and *Desulfovibrio* at the genus level were higher. These results show that gut microbiota is dysregulated in GDM patients (Crusell et al., 2018). Higher bacterial richness and association with metabolic and inflammatory variables were detected throughout pregnancy in GDM patients (Ferrocino et al., 2018). Compared with normal pregnancies, GDM patients did have an altered gut microbiota composition. This also suggests that the approaches to improving the gut microbiota are potential directions to influence the metabolic-related health of the mother.

With the change of gestational age, on the one hand, the gut microbiota is involved in the physiological adaptation of pregnant women’s metabolism; on the other hand, the abnormal composition of the gut microbiota of pregnant women is related to the higher possibility of GDM and is related to macrosomia, premature birth, and other adverse pregnancy outcomes. Therefore, it has been proposed to modify the gut microbiota for adjuvant treatment or prevention of GDM.

## Dietary supplements mechanisms of action

**Figure 1:**
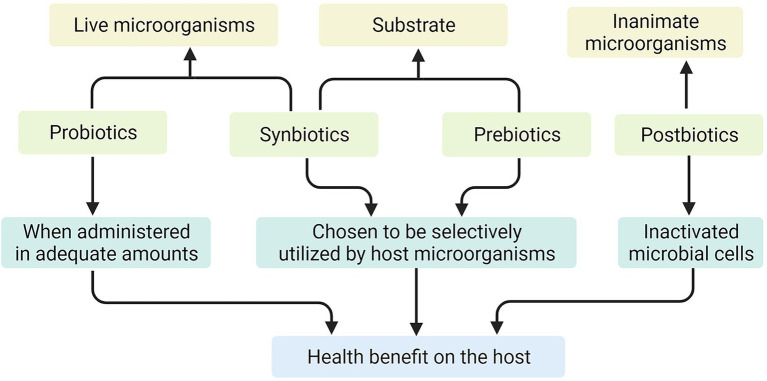
The mechanisms of action of probiotics, prebiotics, synbiotics, and postbiotics.

Probiotics are defined as, “live microorganisms that, when administered in adequate amounts, confer a health benefit on the host” (Hill et al., 2014). Probiotics can directly act on the intestinal mucosal barrier, regulate immune response, thereby increasing glucose tolerance, and may restore the gut microbiota imbalance caused by obesity and diabetes (Gibson et al., 2017). Prebiotics are not digested and absorbed by the host but can promote the metabolism and proliferation of beneficial bacteria in the body selectively and selectively utilized by host microorganisms that confer a health benefit (Marco et al., 2021). They promote the value-added of target flora and improve intestinal microecology by increasing the abundance of probiotics in the intestine (Gibson et al., 2017). Synbiotics are mixture comprising live microorganisms and substrates selectively utilized by host microorganisms that confers a health benefit on the host (Swanson et al., 2020). And postbiotics are defined as, “preparation of inanimate microorganisms and/or their components that confers a health benefit on the host” (Salminen et al., 2021; Figure 1). Most mechanism research on probiotics or prebiotics is based on studies using animals, cell cultures, or *in vitro* human models, some of which are unproven in humans.

Mechanisms of *Lactobacillus* and *Bifidobacterium* specific probiotics are to enhance intestinal epithelial barrier, modulate mucus production, and improve tight junction protein expression (La Fata et al., 2018). Some probiotics may enhance intestinal barrier function by increasing mucus-secreting genes that reduce pathogen-epithelial cell binding (Shen et al., 2018); while factors that improve barrier function also include the downregulation of inflammation (Sanders et al., 2018).

Probiotics also work by promoting the production of short-chain fatty acids (SCFAs) and other small molecular acids. For example, probiotics including *Lactobacillus* and *Bifidobacterium* produce lactic acid and acetic acid as major products of metabolism. SCFAs have particular utility in helping to improve the gut environment. When these SCFAs are made, they lower the pH of the lumen and prevent the pathogen growth (Ríos-Covián et al., 2016). SCFAs can activate cells through the surface of cells G protein−coupled receptors (GPR). For example, SCFAs activate GPR41 and GPR43 on intestinal epithelial cells (Brown et al., 2003). SCFAs and GPR41/43 promote acute inflammatory responses in the intestine for tissue inflammation and protective immunity (Kim et al., 2013). SCFAs also regulate glucose and lipid metabolism through GPR41/43. Most SCFAs are absorbed by colon cells and liver as their energy source, while others are metabolized by muscle and adipose tissue (van der Beek et al., 2015). Prebiotics is commonly consumed to improve health by stimulating the growth of beneficial bacteria and the production of SCFAs. One study analyzed the effects of five widely used prebiotic fibers on beneficial bacteria and SCFAs, and found intaking prebiotic fibers promoted the formation of beneficial SCFAs (Carlson et al., 2017).

Some probiotics have been shown to interact directly with dendritic cells, and increase the activity of phagocytic or natural killer cells (Klaenhammer et al., 2012). Levels of anti-inflammatory cytokines can be increased by supplementation with probiotics (Klaenhammer et al., 2012). After prebiotics supplementation, pro-inflammatory cytokines interleukin-6 (IL-6), tumor necrosis factor-α (TNF-α), interleukin-1β (IL-1β) decreased, and anti-inflammatory cytokines interleukin-10 (IL-10) raised (Vulevic et al., 2015). In addition, a randomized placebo-controlled trial of *Lactobacillus rhamnosus* HN001, found it had a significant protective effect against eczema development at 2–6 years of age while preventing allergic sensitization when children were 6 years of age (Wickens et al., 2018).

Prebiotics may also affect satiety healthy. Some SCFAs produced by gut fermentation influence free fatty acid receptor 2 (FFAR2). In addition, SCFAs also regulate the lipolysis and the release of incretin glucagon-like peptide-1 (GLP-1; Bolognini et al., 2019). According to an animal study, SCFAs acid acetate formed by prebiotics goes through the blood–brain barrier and into the hypothalamus, stimulating anorexia signaling and playing a direct role in central appetite regulation (Frost et al., 2014). Several studies have shown that SCFAs acted on colonic epithelial cells to induce the production of anorexia hormones peptide-YY (PYY) and GLP-1 (Christiansen et al., 2018).

Since prebiotics are often indigestible dietary fiber, they are also beneficial for improving gut function. Improvements in fecal characteristics, including defecation frequency and fecal viscosity, have also been observed with prebiotics or probiotics in several randomized controlled trials (Ford et al., 2014; Micka et al., 2017). It has also been observed in animal experiments that SCFAs produced by prebiotic fermentation, such as butyrate and propionate regulate intestinal peristalsis and contraction at different speeds (Hurst et al., 2014). The prebiotic inulin also improves constipation and softens stools (Micka et al., 2017).

**Figure 2:**
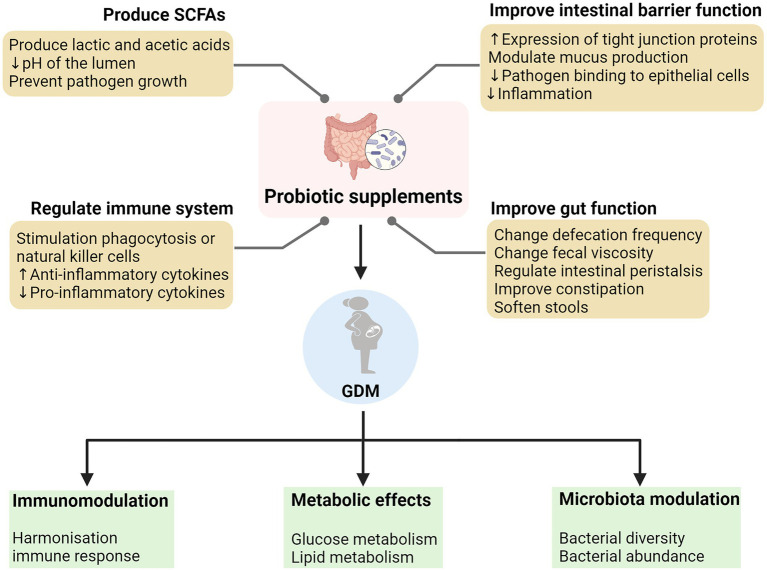
The mechanisms of action of probiotic supplements and its role in alleviating the pathology of gestational diabetes mellitus (GDM).

**Figure 3:**
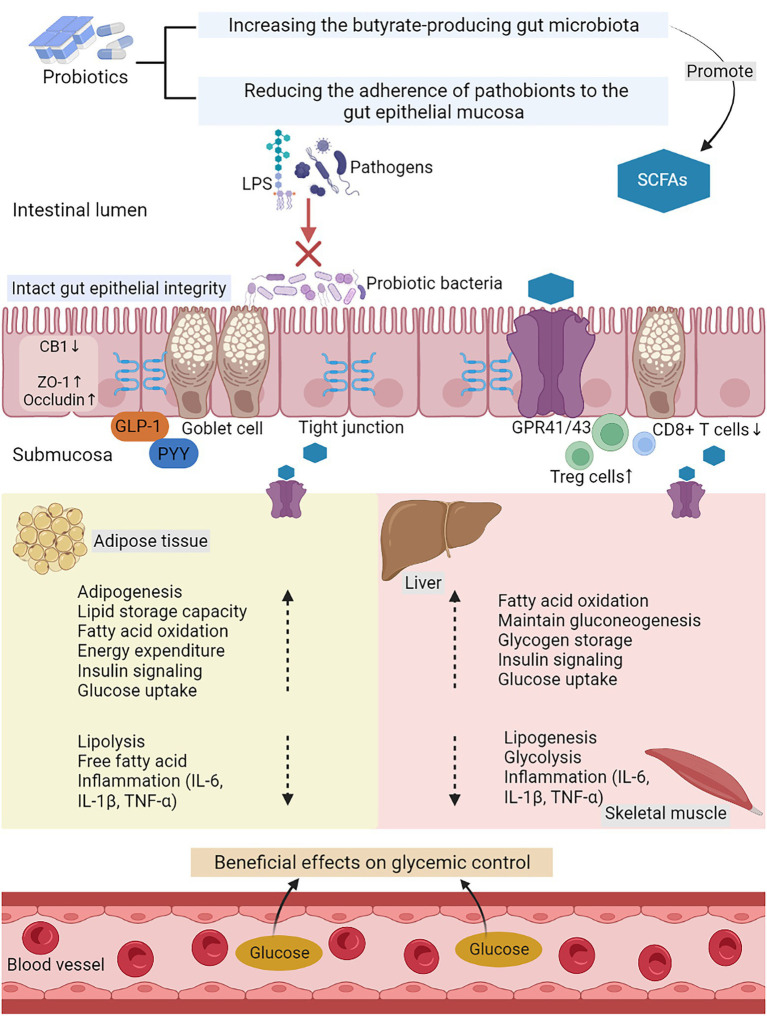
The possible mechanisms of action of probiotics in GDM. LPS, lipopolysaccharide; SCFAs, short-chain fatty acids; GPR 41/43, G-protein-linked receptor 41/43; CB1, cannabinoid receptor 1; ZO-1, zona occludens 1; GLP-1, glucagon like peptide-1; PYY, peptide YY; Treg cells, regulatory T cells; IL-6, interleukin-6; IL-1β, interleukin-1β; and TNF-α, tumor necrosis factor-α.

Probiotics work through a variety of means, including improving the functional integrity of the gut barrier, producing SCFAs, and regulating the immune function (Figure 2). In addition, they also increase satiety and reduce weight. The improvement of intestinal function and constipation also has certain benefits. The use of dietary supplements as an intervention may be a promising approach. The possible mechanisms of action of probiotics in GDM are summarized in Figure 3.

## Prevention and treatment effects of dietary supplements on GDM

### Prevention effects on GDM

Dietary supplements play a role in the prevention and treatment of GDM by regulating the gut microbiota of pregnant women. Probiotics are still controversial in preventing GDM and reducing the incidence of GDM in pregnant women. Wickens et al. (2017) reported in the clinical trial of normal-weight pregnant women that daily supplementation of *L. rhamnosus* HN001 (6 × 10^9^ CFU) during 14–16 weeks of gestation reduced the incidence and recurrence rate of GDM, especially in elderly pregnant women and people who have a history of GDM.

However, the effect of probiotics intervention was inconsistent among high-risk pregnant women, such as overweight and obesity. Shahriari et al. (2021) finally analyzed 507 elderly obese pregnant women taking probiotics between 14 and 24 weeks of gestation. The results showed that the incidence of GDM was 41.9% in the probiotic group and 40.2% in the placebo group, and the difference was not statistically significant (*p* = 0.780). Supplementation of probiotics for a certain period in the second trimester did not decrease the incidence of GDM (Shahriari et al., 2021). A study by Callaway et al. (2019) compared the results of probiotics and placebo groups in obese and overweight pregnant women revealed probiotics containing *Bifidobacterium lactis* and *L. rhamnosus* were not very good at preventing GDM. Similarly, Pellonperä et al. (2019) evaluated whether daily use of fish oil and probiotic supplements in overweight and obese pregnant reduced the risk of GDM and improved the glucose metabolism. It turned out that there was no positive reduction (Pellonperä et al., 2019; Table 1). Therefore, weight and other high-risk pregnancy conditions are the critical factors affecting the preventive effect of probiotics on GDM. Meanwhile, some studies have found changes in the relative abundance of bacterial species during pregnancy in the overweight and obese pregnant women without GDM, suggesting that this population gain more benefit from the dietary regulation of gut microbiota (Mokkala et al., 2021). The presence of GDM may interfere with the flexibility of mother gut microbiota, therefore limiting the ability of GDM patients’ feedback to dietary regulation. Thus, the clinical application of probiotics in high-risk pregnant women needs to be further studied.

**Table 1:** Clinical efficacy of dietary supplements on GDM.

Supplements	Doses	Duration	Sample size	Target disease	Main effects	References
**Probiotics**						
*Lactobacillus rhamnosus* HN001	6 × 10^9^ CFU	14–16 weeks’ gestation	423	Gestational diabetes mellitus	Reduce the incidence and recurrence rate of GDM.	Wickens et al., 2017
*Lactobacillus acidophilus* LA1, *Bifidobacterium longum* sp54 cs, and *Bifidobacterium bifidum* sp9 cs	1.5 × 10^10^ CFU	14–24 weeks’ gestation	542	Gestational diabetes mellitus	Could not reduce the incidence of GDM.	Shahriari et al., 2021
*Bifidobacterium lactis* and *Lactobacillus rhamnosus*	1 × 10^9^ CFU	16–28 weeks’ gestation	411	Gestational diabetes mellitus	GDM cannot be prevented.	Callaway et al., 2019
*Lactobacillus rhamnosus* HN001 and *Bifidobacterium animalis* ssp. lactis 420	1 × 10^10^ CFU	14 weeks’ gestation~6 months postpartum	439	Gestational diabetes mellitus	It could not reduce the incidence of GDM or improve glucose metabolism.	Pellonperä et al., 2019
*Bifidobacterium bifidum* and *Lactobacillus*	2 × 10^9^ CFU	24–28 weeks’ gestation	57	Gestational diabetes mellitus	Significantly improved glucose metabolism, including FPG fasting insulin, and insulin resistance.	Kijmanawat et al., 2019
*Lactobacillus acidophilus, Lactobacillus casei*, *Bifidobacterium bifidum*, and *Lactobacillus fermentum*	2 × 10^9^ CFU/g	6 weeks	48	Gestational diabetes mellitus	It has beneficial effects on the expression of insulin and inflammation-related factors, control of blood glucose, lipid metabolism, inflammatory markers, and oxidative stress.	Babadi et al., 2019
*Lactobacillus acidophilus*, *Lactobacillus casei*, and *Bifidobacterium bifidum*	2 × 10^9^ CFU/g	6 weeks	60	Gestational diabetes mellitus	Beneficial effects on FPG, hs-CRP, TAC, MDA, and oxidative stress index.	Badehnoosh et al., 2018
*Lactobacillus acidophilus*, *Lactobacillus casei*, and *Bifidobacterium bifidum*	2 × 10^9^ CFU/g	6 weeks	60	Gestational diabetes mellitus	Beneficial effects on glycaemic control, triglycerides, and VLDL cholesterol concentrations.	Karamali et al., 2016
*Lactobacillus acidophilus* LA-5, *Bifidobacterium* BB-12, *Streptococcus thermophilus* STY-31, and *Lactobacillus delbrueckii bulgaricus* LBY-27	4 × 10^9^ CFU	8 weeks	64	Gestational diabetes mellitus	Affect glucose metabolism and weight gain.	Dolatkhah et al., 2015
*Lactobacillus salivarius* UCC118	1 × 10^9^ CFU	From GDM diagnosis until delivery	149	Gestational diabetes mellitus	Had no impact on glycemic control.	Lindsay et al., 2015
*Streptococcus thermophilus*, *Bifidobacterium breve*, *Bifidobacterium longum*, *Bifidobacterium infantis*, *Lactobacillus acidophilus*, *Lactobacillus plantarum*, *Lactobacillus paracasei*, and *Lactobacillus delbrueckii* subsp. Bulgaricus	112.5 × 10^9^ CFU	8 weeks	82	Gestational diabetes mellitus	May help to modulate inflammatory markers and may have benefits on glycemic control.	Jafarnejad et al., 2016
**Synbiotics**						
*Lactobacillus probioti*c strains consisting of *L. acidophilus, L. plantarum, L. fermentum,* and *L. gasseri* plus fructooligosaccharide	1.5–7.0 × 10^9–10^ CFU/g and 38.5 mg	6 weeks	90	Gestational diabetes mellitus	There were no significant changes in TAC, FPG, and insulin resistance/sensitivity index.	Nabhani et al., 2018
*Lactobacillus acidophilus*, *Lactobacillus casei*, and *Bifidobacterium bifidum* plus inulin	2 × 10^9^ CFU/g and 800 mg	6 weeks	70	Gestational diabetes mellitus	Beneficial effects on insulin metabolism, TAG, and VLDL-C.	Ahmadi et al., 2016

In brief, whether probiotics prevent GDM in pregnant women remains controversial. For normal-weight pregnant women, certain dietary supplements effectively reduce the risk of GDM. Still, the intervention effect is limited for overweight and obese pregnant women, and body weight is a factor affecting the prevention effect of probiotics. The presence of GDM also interferes with the gut microbiota, reducing the sensitivity of dietary supplements to intervention. The research about the intervention of dietary supplements on intestinal microecology during pregnancy is still in the initial stage and need further improved.

### Treatment of GDM

#### Treatment effects of probiotics on GDM

Some studies suggest that probiotics supplementation in pregnant women with GDM during the second and third trimesters reduces FPG and increases insulin sensitivity, which may be an adjunct therapy to control GDM. Fifty-seven pregnant women with GDM were enrolled at the second trimester of gestation and randomized to intake probiotic supplements containing *Bifidobacteria* and *Lactobacillus* or placebo, the probiotic group showed the benefits of glucose metabolism, including FPG, fasting insulin, and insulin resistance (Kijmanawat et al., 2019). Babadi et al. (2019) conducted a trial investigating the effect of probiotic supplementation on cytokine expression and metabolism in 48 GDM patients. After 6 weeks of continuous intervention, the results showed beneficial effects on the expression of insulin and inflammatory factors, glucose control, lipid metabolism indicators, inflammatory markers, and oxidative stress (Babadi et al., 2019). Similarly, a clinical trial was completed among 60 subjects with GDM. Subjects were randomly allocated to intake probiotic capsules or placebo. And they found probiotic supplements had beneficial effects on FPG, high-sensitivity C-reactive protein (hs-CRP), total antioxidant capacity (TAC), malondialdehyde (MDA), and oxidative stress index (Badehnoosh et al., 2018). Karamali et al. (2016) studied the effects of probiotics intake on blood glucose control and blood lipid levels in GDM women. The results showed that probiotics have beneficial effects on glycaemic control, triglycerides, and very low-density lipoprotein cholesterol (VLDL-C) concentrations (Karamali et al., 2016). A study also examined the effects of probiotics on weight gain and glucose metabolism in GDM. Compared with the placebo group, the probiotics intervention group reduced the fasting blood glucose, weight gain also decreased during the last 2 weeks of the study (Dolatkhah et al., 2015). However, a clinical trial involving 149 GDM patients had no effect on glycemic control after probiotics intervention (Lindsay et al., 2015). Probiotics play a role in immune response and inflammatory regulation. About 82 women diagnosed with GDM were randomly assigned to receive probiotics or placebo for 8 weeks. Women with GDM who received probiotics had significantly reduced levels of TNF-α, hs-CRP, and IL-6 (Jafarnejad et al., 2016; Table 1).

In summary, probiotics improve glucose metabolism, lipid metabolism, and inflammatory markers in GDM. There are also several differences between different studies. Most studies used probiotics containing two or more strains. However, Lindsay et al. used a single strain (*Lactobacillus salivarius* UCC118; Lindsay et al., 2015). The dosage of probiotics was also different, 2 × 10^9^ CFU/g was a commonly used dose. The populations included in these studies ranged from a few dozen to more than 100. The length of intervention time also affects the intervention effects of probiotics. The duration of intervention in these studies was at least 6–8 weeks. Some confounding factors, such as diet patterns and physical activity, also influence the results. In the future, more clinical trials are needed to investigate the efficacy of probiotics in the treatment of GDM.

#### Treatment effects of synbiotics on GDM

Synbiotics have some disputes on insulin metabolism or lipid metabolism in GDM pregnant women. In a study, 90 pregnant women with GDM were supplemented with synbiotics containing *Lactobacillus* and fructooligosaccharides. Compared with the placebo group, FPG, insulin resistance index, insulin sensitivity, lipid mass spectrometry, and TAC in the synbiotics supplemented group had no significant changes (*p* > 0.05; Nabhani et al., 2018). The difference is that Ahmadi et al. and others took synbiotics supplements containing multiple strains and inulin in patients with GDM for 6 weeks, which had beneficial effects on insulin metabolism and VLDL-C (Ahmadi et al., 2016; Table 1).

**Figure 4:**
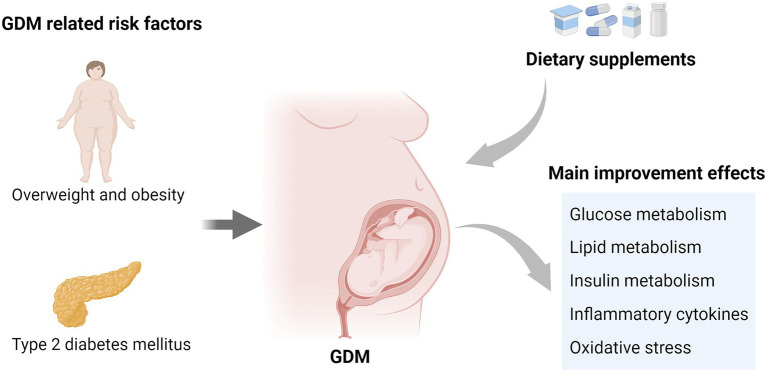
GDM related risk factors and the main improvement effects of dietary supplements on GDM.

In short, there are different conclusions in the studies on the intervention of synbiotics on GDM, and different strain combinations and doses may be the possible reasons for the difference. Clinical studies on different combination preparations are expected. The main improvement effects of dietary supplements on GDM are summarized in Figure 4.

#### Dietary supplements and existing treatment strategies for GDM

The current treatment for GDM is to reverse hyperglycemia and reduce the risk of adverse pregnancy outcomes. An important part of gestational diabetes management is lifestyle interventions including healthy eating, physical activity, weight management, and self-monitoring of blood sugar levels. A Cochrane study evaluated the effect of lifestyle interventions on women with GDM. Exposure to lifestyle interventions reduced the risk of large for gestational age infants (RR 0.60, 95% CI 0.50–0.71). Lifestyle interventions were associated with a reduced risk of postpartum depression in women (RR 0.49, 95% CI 0.31–0.78; Brown et al., 2017). Other studies have shown that specific dietary interventions have a positive impact on maternal blood glucose, fasting blood glucose, and postprandial blood glucose have varying degrees of decline (Yamamoto et al., 2018).

If the lifestyle intervention cannot achieve the goal of blood glucose control, pharmacotherapy is used. Insulin injection therapy is a common method. Metformin and glibenclamide are also used as oral treatment for GDM. A randomized controlled trial was conducted to study the effect of glibenclamide or insulin combined with metformin in the treatment of gestational diabetes. It was found that the combination of metformin and insulin was superior to the combination of metformin and glibenclamide in the control of blood glucose (Reynolds et al., 2017). Affres et al. (2021) showed that glibenclamide was an effective treatment for women with GDM to achieve blood glucose targets during pregnancy. When glibenclamide was not well controlled, blood glucose control was improved by switching to insulin (Affres et al., 2021). Studies of the long-term effects of pharmacological therapy on mothers and fetuses are less clear.

In recent years, the relationship between GDM and diet has promoted the study of dietary supplements as a potential prevention and treatment strategy for GDM. Dietary supplements are well tolerated, safe and easy to administer. Dietary supplements such as probiotics and prebiotics usually produce effect by targeting the gut microbiota. Eating food rich in prebiotic fiber along with fermented foods promotes the growth of bacteria that break down plant starches and fibers into SCFAs. SCFAs may impact the expression of appetite regulation hormones, such as GLP-1 (Tilg and Moschen, 2015). In the second and third trimester of pregnancy, the use of probiotic supplements in women with gestational diabetes decreased fasting blood glucose and increased insulin sensitivity (Kijmanawat et al., 2019). Larger, longer trials of different probiotic strains are needed.

## Effects of dietary supplements on GDM related risk factors

### Overweight and obesity

Pre-pregnancy overweight and obesity are linked to increased GDM risk (Li et al., 2020). A cohort study based on an Australian population also assessed the profound influence of overweight and obesity of GDM, and the results were consistent (Black et al., 2022). Moreover, the increase of BMI before pregnancy is also related to maternal and infant adverse outcomes, such as the risks of large for gestational age (LGA), macrosomia, and cesarean delivery (Zheng et al., 2022). It is meant to improve the overweight and obesity status through dietary supplements before pregnancy. Meanwhile, obesity is closely related to gut microbiota. Changes in gut microbiota are observed in obese people.

Regulating beneficial flora through specific probiotics may be a potential treatment option for obesity (Balakumar et al., 2018). An 8-week clinical trial of 101 obese adolescents showed that the daily intake contained 2 × 10^9^ CFU/AFU *Bifidobacterium breve* BR03 and B632 improved metabolic indexes, insulin sensitivity, reduced body weight, and the number of *Escherichia coli* (Solito et al., 2021). In addition, in a study in Indonesia, 60 overweight adults were fed probiotic powder containing *Lactobacillus plantarum* Dad-13 for 90 days. The results revealed that compared with the placebo group, the weight and body mass index (BMI) of the intervention group significantly decreased, and amounts of *Bacteroidetes*, especially *Prevotella*, increased significantly, while the number of *Firmicutes* decreased significantly (Rahayu et al., 2021). The commonly used probiotics include *Lactobacillus* and *Bifidobacterium* strains. The combination of multiple strains may have an excellent clinical prospect in order to study the recovery of gut microbiota with probiotics, which is a possible clinical target for the treatment of flora-related diseases, such as obesity. Gomes et al. (2020) conducted cluster analysis on the subjects according to their body composition after taking probiotic mixture into 32 overweight or obese women. They found that the proportion of TM_7_ in obese women was higher. At the same time, after the intervention, *Clostridiaceae* increased, and TM_7_ bacteria tended to decrease. Intervention specific bacteria such as TM_7_ bacteria are considered as a new target method for the treatment of obesity (Gomes et al., 2020).

Prebiotic intake also improves overweight and obesity and its adverse effects. A clinical trial of 150 obese people showed that the daily intake of 16 g inulin for 3 months reduced weight to a greater extent than the placebo group by regulating specific flora, and reducing diastolic blood pressure (DBP), aspartate aminotransferase (AST), and insulinemia (Hiel et al., 2020). Reimer et al. conducted a 12 weeks study on overweight or obese people. They found fructooligosaccharides inulin and whey protein was helpful to regulate appetite, but fructooligosaccharides increased the abundance of *Bifidobacterium* (Reimer et al., 2017). Obesity often leads to emotional disorders. Research scholars analyzed the changes in gut microbiota and their effects on emotion and cognition in 106 obese patients treated with prebiotic inulin and placebo. The results showed that inulin supplementation had moderate beneficial effects on emotional ability and cognitive flexibility. In particular, patients with higher levels of *Coprococcus* at baseline were more likely to benefit from prebiotic supplements in terms of mood (Leyrolle et al., 2021). Thus, people with specific microbial characteristics or intestinal types will be more sensitive to prebiotics. In addition, recent evidence suggests that increasing colonic and plasma concentrations of SCFAs protect against obesity and obesity-induced insulin resistance, which is a novel approach (Canfora et al., 2015). Gut microbiota ferment prebiotics, such as inulin into acetate, propionate, and butyrate. In one study, 14 overweight and obese men were given inulin preparation, and the acute metabolic effects after a single dose of 24 g were tested. The results showed that the intake of prebiotic inulin improved fat oxidation, increased the level of metabolites of gut microbiota, and promoted the production of SCFAs (van der Beek et al., 2018).

The use of prebiotics or probiotics alone probably has a better improvement effect, but the synergistic effect of combined use of synbiotics is not clear. Some researchers think that synbiotics are more effective than probiotics alone in correcting the destruction of gut microbiota caused by obesity. Sergeev et al. (2020) supplemented synbiotics to 20 obese patients participating in the weight loss program for 3 consecutive months. As a result, the abundance of intestinal bacteria that have a positive impact on health was increased, especially *Bifidobacteria* and *Lactobacillus*. Synbiotics supplements can regulate human intestinal microorganisms by increasing the abundance of potentially beneficial bacteria (Sergeev et al., 2020). However, Krumbeck et al. compared the effects of prebiotic GOS and probiotic *Bifidobacterium* IVS-1 and *B. lactis* BB-12 when used alone and as synbiotics. The results showed that single-use improved colonic permeability, but there was no synergistic effect when used together (Krumbeck et al., 2018).

In summary, regulating the microbiota through the ingestion of specific probiotics is a new target for treating obesity, regulating the growth of beneficial bacteria while reducing the number of harmful bacteria currently, *Bifidobacteria* and *Lactobacillus* are the most commonly used probiotics. Compared with non-obese people, obese people have different intestinal types and are more sensitive to intervention. The composition of bacteria in obese people is different from that in non-obese people, and regulation of these differences may be a therapeutic direction. Prebiotics improved the adverse symptoms of overweight and obesity. They not only reduce weight by regulating appetite but also reduce blood pressure, improve the negative mood caused by obesity, and improve cognitive performance. Further studies are needed on the synergistic effects of probiotics and probiotics, and strains, dosage forms, and formulations can be enhanced to optimize the combination of these dietary supplements.

### Type 2 diabetes mellitus

Type 2 diabetes mellitus is relative insulin deficiency owing to β-cell dysfunction and insulin resistance (Eizirik et al., 2020). In the GDM population, the risk of subsequent development of T2DM is several times higher than those people with average blood glucose (Vounzoulaki et al., 2020). Pregnant women with type 1 and type 2 diabetes are more possibly to get adverse pregnancy outcomes (Murphy et al., 2021). The risk of such diseases also shows the importance of T2DM intervention and prevention (Vounzoulaki et al., 2020).

Type 2 diabetes mellitus is associated with gut microbiota. T2DM can be effectively managed through the regulation of gut microbiota by probiotics. Firouzi et al. randomized 136 T2DM patients to receive probiotics or placebo and found that glycosylated hemoglobin A1c (HbA1c) in the probiotics group decreased by 0.14%, while that in the placebo group increased by 0.02% (*p* < 0.05), fasting insulin decreased by 2.9 U/ml in the probiotic group and increased by 1.8 U/ml in the placebo group (p < 0.05), probiotics supplementation modestly improved fasting insulin and HbA1c (Firouzi et al., 2017). In addition, a study of 68 patients with T2DM confirmed that specific *Lactobacillus reuteri* strains reduced HbA1c and cholesterol by adjusting the abundance of *Bacteroides* and *Bifidobacteria*, and ADR-3 strain is superior to ADR-1 strain in terms of antihypertensive effect and reducing the number of *Firmicutes* (Hsieh et al., 2018).

Previous studies have shown that dietary fiber, lifestyle intervention, and hypoglycemic drugs metformin have been shown to reduce the incidence rate of T2DM (Wu et al., 2017; Zhao et al., 2018). The effectiveness of these interventions is enhanced by regulating the gut microbiota. A randomized controlled study by Palacios et al. studied 60 adults with BMI ≥ 25 kg/m^2^ and who had diabetes prodromal stage or T2DM in the past 12 months. Participants received a multi-strain probiotic or placebo intervention. The primary and secondary outcome indicators were analyzed at baseline and 12 weeks after the intervention, and the fecal microbiota was analyzed by macrogenomic analysis. The results exhibited that for the subgroup taking metformin, FPG, HbA1c, and insulin resistance decreased, and plasma butyrate concentration increased; Compared with the placebo group, the microbial butyric acid production pathway was abundant in the probiotic group. Therefore, probiotics can be used as an adjuvant of metformin by increasing the production of butyrate, which enhance the management of T2DM (Palacios et al., 2020).

Prebiotics also improve T2DM by regulating gut microbiota disorder. Around 46 T2DM patients showed that 10 g of inulin rich in fructooligosaccharides per day for 2 months benefit for blood glucose, blood lipid levels, also including immune indicators (Dehghan et al., 2016). Fructooligosaccharides inulin also has beneficial effects on improving the level of metabolites of gut microbiota. Birkeland et al. studied 25 T2DM patients supplemented with fructooligosaccharides inulin for 6 weeks. Prebiotics had significant bifurcation and increase fecal SCFAs concentration but did not change the diversity of fecal flora (Birkeland et al., 2020). In addition, more and more attention has been paid to the intervention effect of synbiotic supplements on T2DM patients. Kanazawa et al. recruited 88 obese patients with T2DM and supplemented synbiotics for 24 weeks. The results revealed that *Bifidobacteria* and *Lactobacillus* increased after administration, the relative abundance of *Bifidobacteria* increased, as well as the concentration of acetic acid and butyric acid in feces. However, the inflammatory marker IL-6 did not change much. Thus, synbiotics administration at least partially improves the intestinal environment of obese T2DM patients (Kanazawa et al., 2021).

In short, probiotics regulate the gut microbiota to alleviate the adverse effects of T2DM, such as lowering the biochemical parameters related to glucose metabolism. Different strains have different intervention effects, and some strains show better effects in regulating the flora and improving the phenotype. Probiotics enhance the effectiveness of hypoglycemic drugs and exercise interventions, and the mechanism may be the increase of microbial butyric acid production after probiotics intervention to assist metformin treatment. Prebiotic supplements containing fructooligosaccharides inulin affect glucose and lipid status and immune regulation in T2DM patients. Synbiotics supplementation also improved the adverse intestinal environment and increases the content of beneficial bacteria. Therefore, probiotics, prebiotics, and their components alleviate or improve T2DM to some extent. Still, the specific efficacy of these interventions on diabetes needs to be further studied in clinical trials.

## Conclusion and prospects

Gestational diabetes mellitus is nearly related to the disorder of gut microbiota. Although the role of probiotics, prebiotics, and synbiotics in the prevention of GDM is controversial, several studies suggest that dietary supplements have certain effect in reducing GDM (Wickens et al., 2017). In contrast, other studies prove no benefit (Callaway et al., 2019; Pellonperä et al., 2019; Shahriari et al., 2021). The differences are related to several factors, such as the timing of dietary supplements intervention, differences in probiotic strains, and the level of supplement dosage. Furthermore, the BMI of pregnant women affect whether pregnant women develop GDM and the role of targeting gut microbiota, specific populations benefit more from the current intervention, and the presence or absence of GDM status affects the sensitivity of intervention (Mokkala et al., 2021). For pregnant women with GDM, continued use of dietary supplements also delay the adverse progression of GDM and reduce blood glucose (Kijmanawat et al., 2019). However, dietary supplements also have some beneficial effects on GDM, including improving glucose metabolism, insulin metabolism, and lipid levels. Meanwhile, due to the limited number of trials and sample population, more studies on the pregnancy population are needed in the future.

At the same time, this paper also reviewed the intervention effects of dietary supplements on GDM related risk factors, such as for overweight, obesity, and T2DM. Prebiotics reduce weight by increasing satiation and reducing appetite (Bolognini et al., 2019), and partially improve glucose metabolism indicators of T2DM. It suggests that we should pay attention to not only the prevention and treatment of GDM but also the improvement of overweight and obesity before pregnancy, and intervention measures should be taken to prevent of T2DM in GDM population. In recent years, most studies have focused on probiotics and prebiotics effect of GDM, obesity, and T2DM. Some studies have also been carried out on neurological disorders such as Alzheimer’s disease and major depressive disorder (Rudzki et al., 2019; Tamtaji et al., 2019), and digestive diseases such as nonalcoholic fatty liver disease (Bakhshimoghaddam et al., 2018). Dietary supplements have more or less improved the clinical symptoms of these diseases. In the future, researchers should invest more in researching other conditions, such as digestive system metabolic diseases, allergic diseases, cardiovascular diseases, and so on. Studies on the targeting of probiotics, prebiotics, and other related gut microbiota are still in progress, and a large number of clinical trials are needed in the future.

Several clinical trials indicate regulating the composition of gut microbiota is the direct mechanism for improving a variety of diseases. The overall performance is to increase the abundance of *Bifidobacteria*, *Lactobacillus*, *Bacteroides*, *Prevotella*, and *Clostridium*, and regulate the number of *E. coli*, *Firmicum*, and TM_7_ bacteria to decrease. In addition, probiotics and prebiotics also indirectly improve such diseases by regulating gut microbiota metabolites SCFAs, such as acetic acid, propionic acid, and butyric acid. Increasing intestinal permeability also plays a vital role in improving the overall metabolic system by improving intestinal barrier function. Previous studies have shown that dietary supplements work through various pathways, including improving intestinal barrier function by producing SCFAs and regulating immune function. Further cell and animal experiments are needed to explore the in-depth mechanism of gut microbiota and their regulation of signaling pathways to target diseases and play a beneficial role.

Dietary supplements intervention is a promising strategy with beneficial effects on the gut microbiome. Several different strains of probiotics, particularly *Lactobacillus* and *Bifidobacteria*, or prebiotics that modulate beneficial bacteria, have been shown to improve relevant indicators, demonstrating the importance of dietary supplements for disease prevention and symptom improvement. Due to the beneficial effect of dietary supplements intervention in improving a variety of diseases, the combined intervention effect of multiple strains is better. New processing methods such as the development of enzyme-modified prebiotics and probiotics to enhance beneficial effects, as well as the optimization of the formulation of biogenic preparations, are also worthy of further development to achieve optimal therapeutic effects.

## Author contributions

JW conceived and drafted the manuscript and prepared tables and figures. JM reviewed and modified the manuscript. All authors contributed to the article and approved the submitted version.

## Funding

This work was supported by the Beijing Natural Science Foundation (no. S170002).

## Conflict of interest

The authors declare that the research was conducted in the absence of any commercial or financial relationships that could be construed as a potential conflict of interest.

## Publisher’s note

All claims expressed in this article are solely those of the authors and do not necessarily represent those of their affiliated organizations, or those of the publisher, the editors and the reviewers. Any product that may be evaluated in this article, or claim that may be made by its manufacturer, is not guaranteed or endorsed by the publisher.
